# Detection of thermal shift in cellular Keap1 by protein-protein interaction inhibitors using immunoblot- and fluorescence microplate-based assays

**DOI:** 10.1016/j.xpro.2022.101265

**Published:** 2022-04-01

**Authors:** Sharadha Dayalan Naidu, Dina Dikovskaya, Terry W. Moore, Albena T. Dinkova-Kostova

**Affiliations:** 1 Division of Cellular Medicine, School of Medicine, University of Dundee, Dundee DD1 9SY, UK; 2 Department of Pharmaceutical Sciences, College of Pharmacy, University of Illinois at Chicago, Chicago, IL, USA; 3 Department of Pharmacology and Molecular Sciences and Department of Medicine, Johns Hopkins University School of Medicine, Baltimore, MD, USA

**Keywords:** Cell Biology, Cell culture, Cell-based Assays, Molecular Biology

## Abstract

Pharmacologic inhibition of the protein-protein interaction (PPI) interface of the Keap1:Nrf2 complex, which leads to Nrf2 activation and cytoprotective gene expression, offers a promising strategy for disease prevention and treatment. To facilitate identification and validation of small-molecule Keap1:Nrf2 PPI inhibitors in the cellular environment in a low- and medium-throughput manner, we detail two adapted cellular thermal shift assay (CETSA) protocols, Keap1-CETSA, an immunoblotting-based methodology for detecting endogenous Keap1, and Keap1-Glow CETSA, a microtiter plate assay of overexpressed fluorescently-tagged Keap1.

For an example of the use and execution of this protocol, please refer to Dayalan Naidu et al. (2021).

## Highlights

• Detection of thermal shift of Keap1 by protein-protein interaction (PPI) inhibitors
• Immunoblot-based protocol to monitor target engagement of endogenous Keap1 in cells
• Fluorescence-based protocol for engagement of Keap1-mCherry protein in cell lysates
• Suitable for medium throughput screens for Keap1-Nrf2 PPI inhibitors

## Graphical abstract

**Figure:**
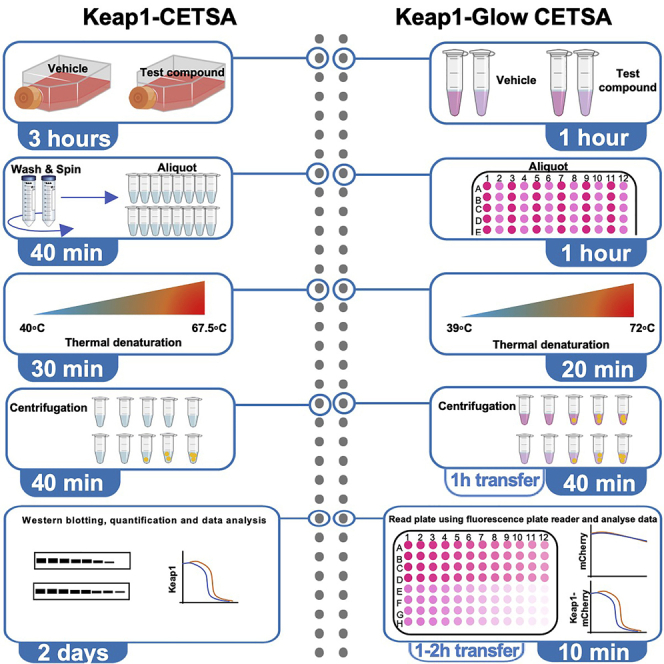

## Before you begin

Small molecules can alter the stability of their target proteins. The ability to monitor and quantify binding of a small molecule to its protein target is vital for target validation and drug development. The cellular thermal shift assay (CETSA) provides a label-free method to monitor target engagement in the physiological environment of the cell. The principle of the CETSA method is based on changes of the thermostability of the target protein upon ligand binding, which is revealed by differential protein denaturation upon exposure to heat. The temperature-induced aggregation (Tm) curve of the protein of interest is generated by monitoring the levels of the protein in the soluble fraction at each selected temperature. The temperature-induced aggregation curve of the protein may be altered upon interaction of the ligand with its cognate protein. Our **Keap1-CETSA** protocol was adapted from Jafari et al. (2014) and could be adapted further for other protein targets. More recently, CETSA coupled with mass spectrometry (CETSA-MS) methods have been developed, which allow for simultaneous evaluation of compound-induced changes in the thermal stability of numerous proteins thus improving throughput (Chernobrovkin et al., 2021). In addition, we have developed a medium throughput microtiter plate-based screening format we have named **Keap1-Glow** **CETSA,** where this assay provides a platform for the identification of small molecules targeting Keap1. To do this, we first generated a cell line where Keap1 ectopically expressed as a fluorescently-tagged doxycycline (Dox)-inducible Keap1-mCherry protein was genomically integrated into human osteosarcoma U2OS cells. As a control, we also generated a U2OS cell line with stably integrated Dox-inducible mCherry protein. The thermostability of the Keap1-mCherry fusion protein or free mCherry protein in lysates obtained from these cell lines is then measured by the fluorescence of the soluble fraction that remains after removal of heat-induced aggregates, over a wide range of temperatures, without the need of performing laborious and time-consuming semi-quantitative western blots.

This article describes two protocols, namely, Keap1-CETSA (Protocol A, steps 1–12) and Keap1-Glow CETSA (Protocol B, steps 13–20). To compare the thermostability profiles of Keap1 in the absence and presence of an inhibitor of the protein-protein interaction (PPI) interface of the Keap1:Nrf2 complex, Keap1-CETSA (Protocol A) uses a western blotting-based technique for detecting endogenous Keap1, and Keap1-Glow CETSA (Protocol B) uses a fluorescence-based microtiter plate assay of overexpressed fluorescently-tagged Keap1.

### For Keap1-CETSA (protocol A)

Ensure that you have cultured an adequate number of HL-60 cells to perform this experiment. As a minimum, 1 × 10^6^ cells, or 100 μg of total protein in a volume of 100 μL, is required for each condition. In this protocol we will be using intact live cells to perform the cellular thermal shift assay (CETSA). The Keap1-CETSA protocol can also be used, with minor modifications, with cell lysates obtained from various cell lines incubated with the vehicle or compound as well as fresh or fresh-frozen tissues collected from animals that had received the vehicle and/or the compound of interest.

### For Keap1-Glow CETSA (protocol B)

The constructs Keap1-mCherry in pcDNA5/FRT/TO and mCherry in pcDNA5/FRT/TO can be cloned or obtained upon reasonable request. These constructs have to be further integrated into a Flp-In^TM^ T-Rex^TM^ host cell line that carries genomically-integrated FRT site and a TET repressor, according to the manufacturer instructions (Flp-In^TM^ T-Rex^TM^ Core Kit, Invitrogen, Cat# K6500-01, https://www.thermofisher.com/order/catalog/product/K650001). Of note, the use of this system results in targeted integration of the constructs into the same locus in every cell, ensuring homogeneous levels of gene expression. For this protocol, this strategy is a powerful tool for expressing efficiently the protein of interest (Keap1-mCherry or mCherry) upon exposure to Dox, overcoming the heterogeneous and unstable expression associated with transient transfection systems. The protocol below describes Keap1-Glow CETSA protocol performed with Flp-In^TM^ T-Rex^TM^ U2OS cells with stably integrated Dox-inducible Keap1-mCherry (KC-U2OS) or mCherry (C-U2OS). It is necessary to use both Keap1-mCherry and mCherry expressing cell lines for this assay.

Expand each of the two cell lines using growth media appropriate for the founder cell type. For standard cell culturing techniques and equipment, please see Phelan and May, 2015 (Phelan and May, 2015, 1.1.1).

For KC-U2OS and C-U2OS, use DMEM containing 10% (v/v) heat-inactivated FBS. Do not add hygromycin B (normally used for selection of cells with integrated constructs) during the expansion of the cells for an experiment. In the last 3–4 days of cell expansion, the media should be supplemented with 0.5 μg/mL doxycycline hyclate to induce expression of the integrated constructs. For each treatment type or control, at least one and a half 15-cm dish or four 10-cm dishes of 85% confluent cells, or 3–4 × 10^7^ cells, is required.

## Key resources table

**Table:** 

REAGENT or RESOURCE	SOURCE	IDENTIFIER
**Antibodies**		

Rat Anti-Keap1 Antibody, clone 144 (1:1,000 dilution)	Sigma-Aldrich	Cat# MABS514
IRDye® 800CW Goat anti-Rat IgG Secondary Antibody (1:20,000 dilution)	LI-COR	Cat# 926-32219

**Chemicals, peptides, and recombinant proteins**		

PRL-295	Terry W. Moore	Lazzara et al. (2020)
Doxycycline hyclate	Sigma-Aldrich	Cat# D9891
Beta-mercaptoethanol	Sigma-Aldrich	Cat# M6250
NuPAGE™ LDS Sample Buffer (4×)	Thermo Fisher Scientific	Cat# NP0007
NuPAGE™ MOPS SDS Running Buffer (20×)	Thermo Fisher Scientific	Cat# NP0001
PageRuler™ Prestained Protein Ladder, 10–180 kDa	Thermo Fisher Scientific	Cat# 26616
cOmplete™, Mini, EDTA-free Protease Inhibitor Cocktail	Roche Diagnostics	Cat# 11836170001
Roswell Park Memorial Institute (RPMI) 1640 cell culture medium for HL-60 cells	Thermo Fisher Scientific	Cat# 11875093
Dulbecco’s Modified Eagle’s Medium (DMEM) for U2OS cells	Thermo Fisher Scientific	Cat# 41966029
Transfer Buffer: 25 mM Tris, 192 mM Glycine, 20% Methanol (v/v)	any	N/A
Phosphate buffered saline (PBS) pH 7.3 ± 0.2 at 25°C: 137 mM NaCl, 2.68 mM KCl, 1.47 mM KH_2_PO_4_, 8.1 mM Na_2_HPO_4_. Stored at 20°C–25°C.	any	N/A
PBST: 0.1% Tween 20 (v/v) in PBS. Stored at 20°C–25°C	any	N/A
PBST-Milk: 5% (w/v) non-fat/skimmed milk powder dissolved in PBST; Stored in the 4°C fridge	any	N/A
Ponceau S stain: 0.1% Ponceau S (w/v) dissolved in 5% (v/v) glacial acetic acid. Stored at 20°C–25°C.	any	N/A

**Experimental models: Cell lines**		

HL-60 cells	ATCC	CCL-240
Doxycycline (Dox)-inducible Keap1-mCherry U2OS (Flp-In™ T-Rex™) cell line (KC-U2OS)	Dayalan Naidu et al., (2021)	N/A
Doxycycline (Dox)-inducible mCherry U2OS (Flp-In™ T-Rex™) cell line (C-U2OS)	Dayalan Naidu et al., (2021)	N/A

**Software and algorithms**		

GraphPad Prism 8.0	GraphPad	https://www.graphpad.com/scientific-software/prism/
Image Studio™ Lite Software	LI-COR	N/A
SoftMax Pro. 7.1	Molecular Devices	N/A

**Other**		

1.5 mL centrifuge tubes (autoclaved)	Sarstedt	Cat# 72.690.001
Serological pipettes	Greiner Bio-One	Cat# 607160
Pipet Buoy	Integra	N/A
Fisherbrand™ Cell Lifters	Fisher Scientific	Cat# 11577692
MicroAmp™ Optical 8-Tube Strip, 0.2 mL	Thermo Fisher Scientific	Cat# 4316567
MicroAmp™ Optical 8-Cap Strips	Thermo Fisher Scientific	Cat# 4323032
Veriti™ 96-Well Fast Thermal Cycler	Thermo Fisher Scientific	Cat# 4375305
Liquid Nitrogen	any	N/A
1.5 L Liquid Nitrogen Dewar	any	N/A
Vortex-Genie 2	Scientific Industries	N/A
Water bath	Grant Instruments	GLS Aqua 12 Plus
50-mL centrifuge tubes	Corning	Cat# 430829
Microcentrifuge, Refrigerated	Thermo Fisher Scientific	MicroCL 17R
Large benchtop centrifuge	Thermo Fisher Scientific	Sorvall Legend T+
Amersham™ Protran® Premium Western blotting membranes, 0.45 μm pore size	Sigma-Aldrich	Cat# GE10600003
NuPAGE™ 4–12%, Bis-Tris, 1.0 mm, Midi Protein Gel, 26-well	Thermo Fisher Scientific	Cat# WG1403BOX
XCell4 SureLock™ Midi-Cell gel electrophoresis system	Thermo Fisher Scientific	Cat# WR0100
PowerPac™ HC High-Current Power Supply	Bio-Rad	Cat# 1645052
Criterion™ Blotter With Plate Electrodes	Bio-Rad	Cat# 1704070
Whatman 3MM CHR Sheets	Sigma-Aldrich	Cat# WHA3030931
SpectraMax M2 microplate reader	Molecular Devices	N/A
Foam floats or racks for water bath to hold 1.5 mL tubes or 96-well rack from standard tip boxes as floats for the 0.2 mL tubes	any	N/A
MicroAmp™ optical 96-well reaction plates (cut to the size to fit the Thermal Cycler)	Thermo Fisher Scientific/ Applied Biosystems	Cat# 4306737
96-well plates, clear: Nunc^TM^ MicroWell^TM^ 96-well, Nunclon Delta-treated flat-bottom microplate	Thermo Fisher Scientific	Cat# 167008
96-well plates, white: Nunc^TM^ MicroWell^TM^ 96 well, Nunclon Delta-treated flat-bottom microplate	Thermo Fisher Scientific	Cat# 136101
Multi-channel pipette	any	N/A
Heat block/heated shaker	any	N/A

## Step-by-step method details

### Protocol A. Keap1-CETSA

In this section of the Keap1-CETSA protocol we will be using intact live cells that have been treated with either vehicle or the Keap1-Nrf2 protein-protein interaction inhibitor PRL-295 (Lazzara et al., 2020) for 3 h to perform the cellular thermal shift assay (CETSA). This protocol can also be adapted for use with lysates derived from cells with a protein concentration of at least 1 mg/mL. In the case of the latter, cell lysates are prepared and incubated with the compound of interest for 1–3 h. Aliquots of the cell lysate are then heated at various temperatures. The use of intact cells over cell lysates has the advantage that it takes into account the ability of the compound to cross the cell membrane.

#### Cell culture and treatment of cells with compound

**Timing: 4–5 h**

1. Prepare the test compounds and thermocycler.
  a. Set a water bath to 25°C. This will be used for the cell lysate preparation steps. Prepare a float/container that can hold PCR strip tubes.
    ***Note:*** We use the plastics from the standard 96-well pipette tip racks from the tip boxes as a float for the tubes.
  b. Warm the cell culture medium to 37°C.
  c. Prepare stock solutions of the compounds, for example by dissolving in an organic solvent that is suitable for the compound of interest and dilute in the cell culture medium at a concentration that can be tolerated by the cells; for this protocol the PPI inhibitor PRL-295 was dissolved in dimethyl sulfoxide (DMSO). To avoid potential cytotoxicity, keep the concentration of the solvent when treating intact cells at 0.1% (v/v).
  d. Pre-program two protocols for the thermal cycler, with six temperature zones each (one with 40°C, 42.5°C, 45°C, 47.5°C, 50°C and 52.5°C, and another, with 55°C, 57.5°C, 60°C, 62.5°C, 65°C and 67.5°C) for 3 min, followed by 25°C for all for a further 3 min.
    ***Note:*** Two protocols are required due to space limitation in the thermocycler as it only contains six VeriFlex blocks which are independent temperature zones.

2. Count the HL-60 cells, adjust to a cell density of 1–2 × 10^6^ cells per mL, and plate 15 mL of cell suspension into each of six separate T75 flasks.

***Note:*** This step could be applied for adherent cells, however, the cells have to be trypsinized following incubation with the test compound to obtain a cell suspension which will then be subjected to heating.

**CRITICAL:** It is important to perform the experiment with the number of cells stated to be able to pellet the aggregated proteins in the subsequent steps.

3. To each of the first three flasks, add 15 μL of vehicle [DMSO used in the example below; final solvent concentration of 0.1% (v/v)]. To each of the other three flasks, add 15 μL of test compound [in this example PRL-295 stock concentration: 10 mM, final concentration 10 μM, is used]. Gently pipette the cell suspension to ensure that the solvent or the compound has been homogeneously mixed.
4. Incubate the cells in a humidified 37°C with 5% CO_2_ incubator for a period of 1–3 h. The incubation time can vary depending on the compound of interest. For this protocol we treated the cells with either the vehicle or compound for 3 h.

#### Cell suspension preparation and heat treatment

**Timing: 2–4 h**

5. Prepare the cell suspension for heat treatment.
  a. Transfer the cell suspensions from each treatment group into their respectively labeled 50 mL centrifuge tubes.
  b. Pellet the cells by centrifugation at 300 × *g* for 4 min at temperature between 20°C-25°C.
  c. Aspirate the supernatant and gently flick the bottom of the tube to loosen the cell pellet before resuspending the cells in 20 mL of PBS.
    **CRITICAL:** It is important to wash the cells with PBS to remove the serum present in the culture media as inadequate removal of the serum can affect the outcome of the immunoblotting process.
  d. Pellet the cells by centrifugation at 300 × *g* for 4 min at temperature between 20°C-25°C. Repeat steps 5.c and 5.d once more.
  e. During the centrifugation process, prepare 10 mL of PBS supplemented with the protease inhibitor cocktail (PBS-PIC).
    ***Note:*** Only 9 mL of PBS-PIC is required, however, to account for losses during pipetting, 10 mL of solution is prepared.
    20× Protease Inhibitor Cocktail (PIC) Stock – Prepared on the day of experiment and kept at 4°C

**Table:** 

Reagent	Final concentration	Amount
Protease Inhibitor Cocktail	20×	1 tablet
PBS	N/A	500 μL
    PBS with Protease Inhibitor Cocktail (PIC) (PBS-PIC) – Prepared on the day of experiment and kept at 20–25°C

**Table:** 

Reagent	Final concentration	Amount
20× Protease Inhibitor Cocktail (PIC) Stock	1×	500 μL
PBS	N/A	9.5 mL
  f. Once the cells have been pelleted, aspirate all the supernatant carefully and gently resuspend the pellet in each tube with 1.5 mL of PBS-PIC and ensure that the cell suspension is homogeneous with no visible clumps.
    ***Note:*** Flick the tube containing the cell pellet gently to loosen it before resuspending in PBS-PIC.

**Figure 1:**
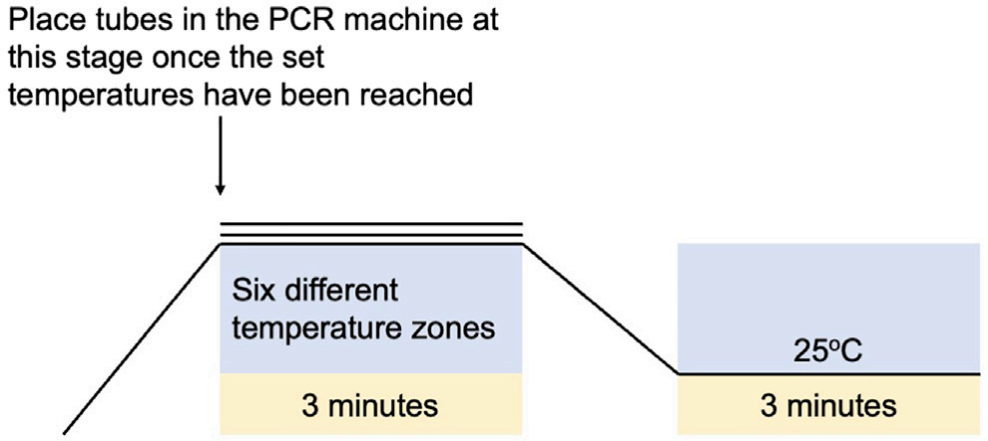
Scheme of the thermocycler setup used for subjecting cells or cell lysates to heat treatment

6. Subject the cells to heat treatment.
  a. Aliquot the cell suspension from each treatment, by transferring 100 μL (∼1–2 million cells) into each of 12 separate 0.2 mL PCR tubes and place the caps onto the tubes.
  b. Label the tubes according to their treatment and temperature (40°C–67.5°C, 2.5°C increments).
  c. Start the program on the thermal cycler that you have set up previously (step 1.d) and ensure that the temperature gradient has been established before placing the first set of tubes (40°C–52.5°C, 2.5°C increments) into the pre-heated thermocycler as shown below. Place the tubes in the thermocycler once the temperature has been reached (Figure 1).
    **CRITICAL:** Do not place the tubes in the thermal cycler during the temperature ramp-up stage to avoid exposure to incorrect temperatures.
  d. Once the program has ended, remove the tubes from the PCR machine and let the tubes cool at temperature between 20°C-25°C for a further 3 min.
  e. Snap-freeze the tubes from step 6.d in liquid N_2_.
  f. Repeat steps 6.c and 6.d for the next set of tubes with the second thermocycler program (55°C–67.5°C, 2.5°C increments).
  g. Snap-freeze the tubes from step 6.f in liquid N_2_.
    **CRITICAL:** Wear protective goggles and gloves when transferring tubes from liquid N_2_ as there is a possibility that the caps may pop out or the strip tubes may crack due to the extreme differences in temperature. To prevent this from happening, ensure that the container you are transferring your samples to is pre-chilled. Use forceps to transfer tubes from liquid N_2_ to prevent bodily injuries.
    **Pause point:** The experiment can be paused at this point by transferring the tubes to the −80°C freezer for storage.

7. Obtain the soluble fraction from the heat-treated cells and prepare lysates for immunoblotting analysis.
  a. Remove the tubes from liquid N_2_ (or take out the tubes that have been stored in the −80°C freezer), place on a 96-well plastic float, and transfer to the 25°C water bath to thaw. Check the samples every minute until the samples are completely defrosted.
    ***Note:*** It usually takes 3–4 min for the samples to completely thaw. We use the plastic racks from standard 96-well pipette tip boxes as a float for the 0.2 mL tubes.
  b. Once the lysates have thawed, vortex briefly with a few pulses before freezing them in liquid N_2_.
  c. Repeat the freeze-thaw cycle (steps 7a and 7b) three more times.
  d. Transfer all the lysate (∼100 μL) from each tube into a labeled 1.5 mL microcentrifuge tube.
  e. Place all tubes into a centrifuge and spin the lysates at 17,000 × *g* for 40 min at 4°C.
  f. After the centrifugation has ended, carefully place all tubes on ice.
    **CRITICAL:** Ensure that the ice in the bucket is not compacted. Transfer the tube from the centrifuge to the ice bucket very carefully to prevent the possibility of dislodging the pellet containing the cell debris and protein aggregates. Alternatively, use cold blocks (which fit 1.5 mL tubes) placed on ice.
  g. Carefully pipette 60 μL of the lysate from each tube without touching the pellet and sides of the tubes and transfer it into a fresh centrifuge tube containing 20 μL of the 4× LDS sample loading buffer and 4 μL of β-mercaptoethanol (β-ME).
    ***Note:*** SDS sample loading buffer could also be used as an alternative to LDS sample loading buffer. The reducing agent dithiothreitol (DTT) could be used instead of β-ME.
  h. Briefly vortex the tubes to mix all the contents in the tube and leave the tubes at temperature between 20°C-25°C for 30–60 min before gel loading.
    ***Note:*** Although typical gel electrophoresis protocols include a step where the denatured protein samples are heated to 95°C or boiled, we omit this step because it may cause uneven evaporation and, for some proteins, aggregation that interferes with the ability of the proteins to uniformly enter the gel.
    **Pause point:** The experiment can be paused at this point by transferring the tubes to the −80°C freezer for storage after incubation with the reducing agent, β-ME.

#### Immunoblotting analysis

**Timing: 2 days**

8. Resolve the proteins in the soluble fractions using electrophoresis.
  a. Load 13 μL of the samples into each well of a 26-well 4%–12% NuPAGE^TM^ BT midi gel along with pre-stained molecular weight markers using the NuPAGE midi gel running tank using the NuPAGE^TM^ 1× MOPS running buffer. Example sample loading sequence: Molecular weight (MW) marker, DMSO treated samples (12 samples from 40°C-67.5°C) and PRL-295-treated samples (12 samples from 40°C-67.5°C).
  b. Run the gel at 100 V for approximately 2 h 10 min and stop the run once the LDS loading dye reaches the bottom of the gel.

**CRITICAL:** The timing given is a guide and can vary with different gel running systems, therefore, it is important to frequently check the gel while it is running to ensure that the samples are separating well and to observe the dye front to monitor the progress of the electrophoresis.

**Figure 2:**
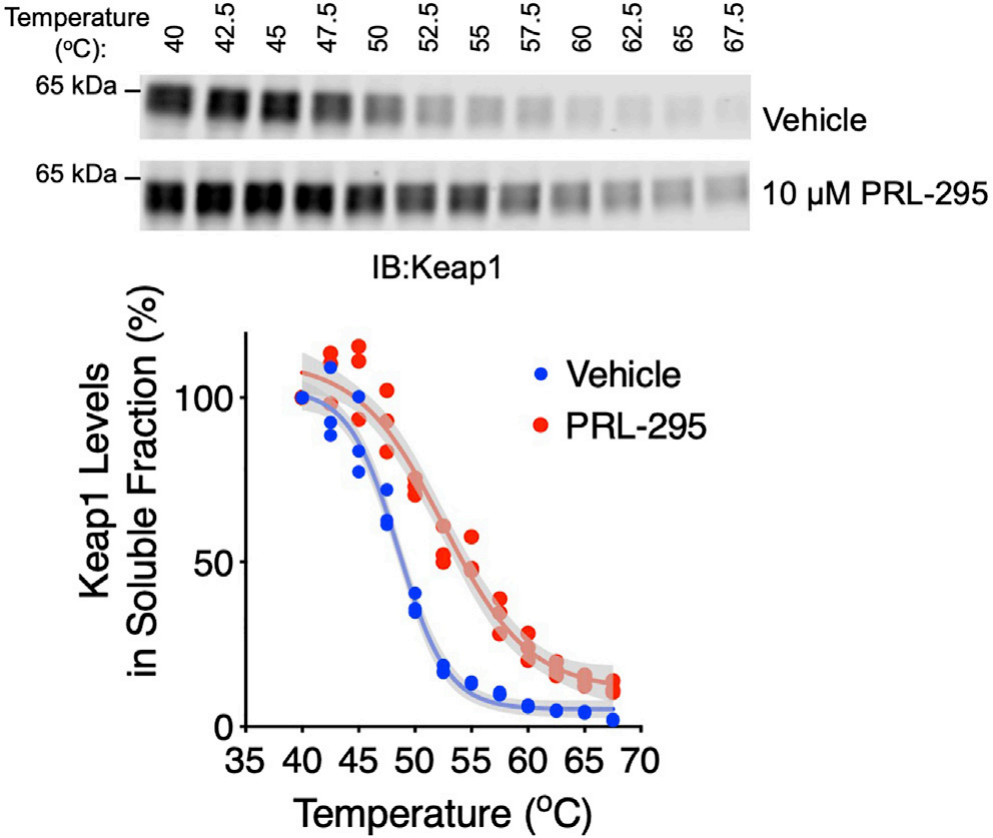
An example of data obtained with Keap1-CETSA (Protocol A) showing that PRL-295 increases the thermostability of Keap1 in intact live HL-60 cells The top panel is a representative immunoblot of Keap1, where the Keap1-CETSA assay was performed using intact live HL-60 cells (1 × 10^6^ cells per mL) treated for 3 h with 0.1% (v/v) DMSO or 10 μM PRL-295. In the bottom panel, each data point (the soluble fraction obtained from 1 × 10^6^ cells) represents the fluorescence intensity of Keap1 normalized to the 40°C treatment from each condition. Shown are individual data points for three replicates for each condition. The data were plotted using GraphPad Prism 8.0 software, where the four parameter logistic (4PL) regression model was used to generate a sigmoidal curve represented with mean and error with 95% confidence interval (CI). Data from Dayalan Naidu et al., 2021.

***Note:*** For this immunoblotting protocol, any western blotting system can be used. For more detailed information on gel electrophoresis and electrophoretic transfer procedure, instructions from the manufacturer Bio-rad can be found at https://www.bio-rad.com/sites/default/files/webroot/web/pdf/lsr/literature/Bulletin_2895.pdf. We have only tested the fluorescently conjugated secondary antibodies (step 10.d) to measure the fluorescence intensities of the band and we suggest using this technique to measure the intensities. We have not tested the chemiluminescence-based detection method for this protocol.

9. Transfer the resolved proteins from the gel to a nitrocellulose membrane.
  a. Once the run has completed, place the gel into the transfer sandwich cassette where it is placed on the top of two pieces of Whatman 3MM filter paper and a sponge, both of which have been soaked in transfer buffer (see key resources table) at the cathode side. Use a roller to ensure that there are no bubbles trapped under the gel.
  b. Place a 0.45-μm nitrocellulose (NC) membrane that has been pre-soaked in transfer buffer on the top of the gel and use the roller to remove any bubbles trapped between the gel and the membrane.
  c. Place two more pieces of transfer buffer-soaked filter paper on top of the NC membrane followed by a sponge which is placed towards the anode side of the cassette.
    ***Note:*** Use the roller to remove any bubbles trapped between the gel and the membrane right before securing the clasp of the transfer cassette.
  d. Fit the transfer sandwich cassette in the Criterion Blotter transfer tank and insert an ice pack, fill the tank up with transfer buffer to the marked fill level and transfer at 70 V for 1 h 10 min at temperature between 20°C-25°C.
  e. Once the transfer is complete, place the NC membrane in a clean container containing deionized water to rinse the blot briefly.
  f. Stain the NC membrane with Ponceau S stain (see key resources table) and rinse with deionized water to visualize the transfer quality.
  g. To remove the Ponceau S stain, wash the NC membrane with PBST for 3 min twice or until the stain has been completely removed.
10. Detect Keap1 on the membrane by immunoblotting.
  a. Block the NC membrane in PBST-Milk for 1 h at temperature between 20°C-25°C.
  b. Incubate the NC membrane for 16–20 h in the Keap1 primary antibody diluted 1:1,000 in PBST-Milk at 4°C.
  c. Wash the NC membrane in PBST for 10 min and repeat this wash step twice.
  d. Incubate the NC membrane in the Goat anti-Rat IRDye 800 secondary antibody diluted 1:20,000 in PBST-Milk for 1 h at temperature between 20°C-25°C, protected from light.
  e. Wash the NC membrane in PBST for 10 min at temperature between 20°C-25°C, protected from light, and repeat this wash step two more times.
  f. Scan the NC membrane using the Odyssey CLx scanner to visualize the bands corresponding to the Keap1 protein (∼65 kDa).
  g. Measure the intensity of each band using the image analysis software Image Studio^TM^ Lite and export the data to an excel spreadsheet.
  h. In each group, normalize the intensity of the bands by setting the intensity of the band for the 40°C treatment to 100% and plot the values (relative to the 40°C treatment intensity) to form aggregation curves for the vehicle treated samples and the compound treated samples. Compare the two curves to observe whether there are differences in the thermostability profile of Keap1. An example of such plot is shown in Figure 2.

#### Quantification and statistical analysis for Keap1-CETSA

11. Quantify the amount of Keap1 in each lane.
  a. Obtain the fluorescence intensity of each band on the western blot image using the ‘Add Rectangle’ or ‘Draw Rectangle’ function found within the Analysis tab in the Image Studio^TM^ Lite software.
  b. After obtaining the signal intensities of all the desired bands, either export the file or copy and paste the intensities (‘Signal’ column) found within the ‘Shapes’ tab into a Microsoft Excel file.
  c. Label the treatment and temperature conditions of each of the acquired band intensities.
  d. Normalize the band intensity from each treatment to its respective 40°C temperature condition where the 40°C band intensity is set to 100%.
12. Using the Prism software, plot the normalized band intensity of Keap1 at each temperature and perform statistical analysis.
  a. Make the data table by choosing a XY Table Format where you will select ‘Enter or import data into a new table’. Click ‘Show the row titles’, where the x values (Numbers) are entered [Temperatures (^o^C)] and the y values [normalized band intensities (%)] are entered as 3 replicate values in side-by-side columns.
  b. Plot the relative band intensities calculated in Microsoft Excel on the y-axis from each temperature condition (x-axis) using the Prism software.
  c. View the graph made by software and under Analysis, choose the ‘Interpolate a standard curve’ option. To select a model, under ‘Standard curves to interpolate’ choose the ‘Sigmoidal, 4PL, X is log(concentration)’ equation with ‘No special handling for outliers’ and plot curve with ‘95% confidence bands’.
  d. A graph showing the curves generated for each treatment (DMSO or 10 μM PRL-295) will be displayed, where an example of such a plot is shown in Figure 2.
  e. To determine statistical significance, from each temperature condition, compare the means of the treatment groups by performing a Student’s t-test.

***Note:*** There are various softwares and methods to analyze and plot the data, this is one example of how the data can be displayed.

### Protocol B. Keap1-Glow CETSA

In this protocol, the lysates of untreated cells expressing fluorescent Keap1-mCherry protein (KC-lysates) or a control mCherry protein (C-lysates) (see section “before you begin”) will be used for treatment with compounds/vehicle, followed by exposure to a temperature gradient, removal of aggregates and fluorescence-based measurements of the remaining soluble fractions.

#### Preparation of cell pellets and thermocycler

**Timing: 1 h**

13. Once the cells are expanded and induced with doxycycline, prepare the cell pellets as follows:
  a. Wash each 15-cm dish thrice with PBS.
  b. Aspirate the PBS and add 1 mL of PBS-PIC (PBS containing 1× Protease Inhibitor Cocktail; also see step 5e for PBS-PIC recipe).
  c. Scrape the cells from each dish with a cell lifter and collect into 1.5 mL centrifuge tube.
  d. Pellet the cells at 400 × *g* for 5 min using a microcentrifuge.
  e. Aspirate the supernatant, leaving a very small amount of it (less than 10 μL) still in the tube.
  f. Snap-freeze the tubes with the cell pellets in liquid nitrogen (N_2_) and store at −80°C until use.

***Note:*** Steps 13.a–e are performed at temperature between 20°C-25°C. Large quantity of frozen cell pellets could be prepared in advance for a large-scale experiment or a series of experiments for added consistency.

14. Pre-program two protocols for the thermal cycler, with six temperature zones each (one with 39°C, 42°C, 45°C, 48°C, 51°C and 54°C and another with 57°C, 60°C, 63°C, 66°C, 69°C and 72°C) for 3 min, followed by 25°C for all for a further 3 min. Of note, two protocols are required due to space limitation in the thermocycler.

#### Cell lysis, compound treatment, heat treatment, and fluorescence measurement

**Timing: 1 day**

15. Prepare for cell lysis.
  a. Set a water bath to 25°C. This will be used for the cell lysate preparation steps.
  b. Pre-warm a thermal shaker to 37°C. This will be used for the incubation of the lysate with the compounds.
  c. Prepare PBS-PIC, 1.5 mL per each cell pellet from a 15-cm dish.
    ***Note:*** If using cell culture dishes other than 15-cm diameter, scale the PBS-PIC amount proportionally to the surface area of the dish.
  d. Prepare a container with liquid N_2_.
  e. Set up the temperature of two microcentrifuges, one to 22°C and another, to 4°C.
16. Prepare cell lysates.
  a. Thaw the appropriate number of cell pellets required for the size of experiment (approximately one and a half pellet from a 15-cm dish or 3–4 × 10^7^ cells of each cell type is required for each treatment or control).
  b. Add 1.5 mL of PBS-PIC to each tube, resuspend well and separate into two 1.5 mL centrifuge tubes, approximately 750 μL each.
    ***Note:*** If using cell culture dishes other than 15-cm diameter, scale the PBS-PIC amount proportionally to the surface area of the dish.
  c. Snap-freeze the tubes in liquid N_2_ and leave in liquid N_2_ for at least 3 min.
  d. Place the samples into a foam float or a rack and transfer it to the 25°C water bath. Thaw for 4–8 min, or until completely thawed.
  e. Vortex.
  f. Repeat steps 16.c–e three more times.
    **Pause point:** The experiment can be paused before the last thawing step, and the tubes could be stored at −80°C for later use. In this case, on the day of continuing the experiment, repeat steps 16.d and 16.e before proceeding.
  g. Spin the tubes at 9,600 × *g* for 5 min at temperature between 20°C-25°C.
  h. Collect the supernatants, combining the supernatants from the same cell type together.
  i. Separate supernatants into 650 μL aliquots, 3 aliquots of each cell type for each treatment or control.
17. Treat the cell lysates with compounds.
  a. Prepare solutions of compounds of interest or vehicle such as DMSO, in PBS at concentration that is 131-fold higher than the desired final concentration, with the final volume in excess of 30 μL for each type of treatment or control.
  b. Mix 5 μL of the compound solution prepared in step 17.a, or 5 μL of vehicle solution, with the 650 μL cell lysate in triplicates, for both cell types.
  c. Incubate at 37°C for 1 h.
18. Subject the treated cell lysates to heat treatment.
  a. Separate each mixture into 12 of 50-μL aliquots, into either 0.2 mL PCR tubes or individual wells of MicroAmp Optical 96-well reaction plates cut to be compatible with the thermal cycler. The pattern should correspond to the temperature zones in the thermal cycler to ensure that each aliquot from the same sample is heated at a different temperature (see example below). Please note that for the settings described here, two plates are required to accommodate the full range of temperatures.
    Example of sample pattern in the heating plates for Keap1-Glow CETSA.
    **Table:** Plate 1

39°C		42°C		45°C		48°C		51°C		54°C	
KC1-V-39	C1-V-39	KC1-V-42	C1-V-42	KC1-V-45	C1-V-45	KC1-V-48	C1-V-48	KC1-V-51	C1-V-51	KC1-V-54	C1-V-54
KC2-V-39	C2-V-39	KC2-V-42	C2-V-42	KC2-V-45	C2-V-45	KC2-V-48	C2-V-48	KC2-V-51	C2-V-51	KC2-V-54	C2-V-54
KC3-V-39	C3-V-39	KC3-V-42	C3-V-42	KC3-V-45	C3-V-45	KC3-V-48	C3-V-48	KC3-V-51	C3-V-51	KC3-V-54	C3-V-54
KC1-D-39	C1-D-39	KC1-D-42	C1-D-42	KC1-D-45	C1-D-45	KC1-D-48	C1-D-48	KC1-D-51	C1-D-51	KC1-D-54	C1-D-54
KC2-D-39	C2-D-39	KC2-D-42	C2-D-42	KC2-D-45	C2-D-45	KC2-D-48	C2-D-48	KC2-D-51	C2-D-51	KC2-D-54	C2-D-54
KC3-D-39	C3-D-39	KC3-D-42	C3-D-42	KC3-D-45	C3-D-45	KC3-D-48	C3-D-48	KC3-D-51	C3-D-51	KC3-D-54	C3-D-54
    **Table:** Plate 2

57°C		60°C		63°C		66°C		69°C		72°C	
KC1-V-57	C1-V-57	KC1-V-60	C1-V-60	KC1-V-63	C1-V-63	KC1-V-66	C1-V-66	KC1-V-69	C1-V-69	KC1-V-72	C1-V-72
KC2-V-57	C2-V-57	KC2-V-60	C2-V-60	KC2-V-63	C2-V-63	KC2-V-66	C2-V-66	KC2-V-69	C2-V-69	KC2-V-72	C2-V-72
KC3-V-57	C3-V-57	KC3-V-60	C3-V-60	KC3-V-63	C3-V-63	KC3-V-66	C3-V-66	KC3-V-69	C3-V-69	KC3-V-72	C3-V-72
KC1-D-57	C1-D-57	KC1-D-60	C1-D-60	KC1-D-63	C1-D-63	KC1-D-66	C1-D-66	KC1-D-69	C1-D-69	KC1-D-72	C1-D-72
KC2-D-57	C2-D-57	KC2-D-60	C2-D-60	KC2-D-63	C2-D-63	KC2-D-66	C2-D-66	KC2-D-69	C2-D-69	KC2-D-72	C2-D-72
KC3-D-57	C3-D-57	KC3-D-60	C3-D-60	KC3-D-63	C3-D-63	KC3-D-66	C3-D-66	KC3-D-69	C3-D-69	KC3-D-72	C3-D-72

The sample names are abbreviated as follows: first 1–2 letters indicate cell type (**KC** for cells expressing Keap1-mCherry, **C** for cells expressing mCherry), followed by a replica number (out of **1, 2 or 3**), the next letter (after dash) indicates treatment (**D** for drug/compound, **V** for vehicle) and the last two numbers (after dash) correspond to the heating temperature. The temperature zone at each position is indicated at the top.
  b. Start one of the pre-programmed protocols (39°C–54°C) on the thermal cycler and ensure that the temperature gradient has been established before placing the set of tubes into the preheated thermal cycler.
    **CRITICAL:** Do not place the tubes in the thermal cycler during the temperature ramp-up stage.
  c. Once the program has ended, remove the tubes/plate from the PCR machine and let them cool at temperature between 20°C-25°C for a further 3 min.
  d. Repeat steps 17.a–c for the next set of tubes/plate, using a second program (57°C–72°C).
19. Determine the amount of Keap1-mCherry and mCherry in the soluble fraction of the heated lysates by measuring the mCherry fluorescence.
  a. Transfer the lysates into pre-labeled 1.5 mL microcentrifuge tubes.
  b. Centrifuge the tubes at 17,000 × *g* for 40 min at 4°C.
  c. Move the tubes onto ice and transfer 40 μL of each lysate into a separate well of a 96-well clear plate (“holding” plate), in the pattern desired for measurements (see 19.d for an example).
    **CRITICAL:** Do not touch the pellet and the side of the tube with the pelleted lysates.
  d. Using a multi-channel pipette, fill white flat-bottom 96-well plate(s) with 65 μL (for KC- lysates) or 85 μL (for C-lysates) of PBS-PIC in a pattern desired for measurements (see example below).
    **CRITICAL:** Include a row of “blanks” with 100 μL of PBS-PIC.
    Example of sample pattern in the measuring plates for Keap1-Glow CETSA.
    **Table:** Plate 1

KC1-V-39	KC1-V-42	KC1-V-45	KC1-V-48	KC1-V-51	KC1-V-54	KC1-V-57	KC1-V-60	KC1-V-63	KC1-V-66	KC1-V-69	KC1-V-72
KC2-V-39	KC2-V-42	KC2-V-45	KC2-V-48	KC2-V-51	KC2-V-54	KC2-V-57	KC2-V-60	KC2-V-63	KC2-V-66	KC2-V-69	KC2-V-72
KC3-V-39	KC3-V-42	KC3-V-45	KC3-V-48	KC3-V-51	KC3-V-54	KC3-V-57	KC3-V-60	KC3-V-63	KC3-V-66	KC3-V-69	KC3-V-72
KC1-D-39	KC1-D-42	KC1-D-45	KC1-D-48	KC1-D-51	KC1-D-54	KC1-D-57	KC1-D-60	KC1-D-63	KC1-D-66	KC1-D-69	KC1-D-72
KC2-D-39	KC2-D-42	KC2-D-45	KC2-D-48	KC2-D-51	KC2-D-54	KC2-D-57	KC2-D-60	KC2-D-63	KC2-D-66	KC2-D-69	KC2-D-72
KC3-D-39	KC3-D-42	KC3-D-45	KC3-D-48	KC3-D-51	KC3-D-54	KC3-D-57	KC3-D-60	KC3-D-63	KC3-D-66	KC3-D-69	KC3-D-72
PBS-PIC	PBS-PIC	PBS-PIC	PBS-PIC	PBS-PIC	PBS-PIC						

Sample abbreviations as in 18.a.

For all rows except the last one, add 65 μL of PBS-PIC and 35 μL of indicated sample.

For the last row, add 100 μL of PBS-PIC.
    **Table:** Plate 2

C1-V-39	C1-V-42	C1-V-45	C1-V-48	C1-V-51	C1-V-54	C1-V-57	C1-V-60	C1-V-63	C1-V-66	C1-V-69	C1-V-72
C2-V-39	C2-V-42	C2-V-45	C2-V-48	C2-V-51	C2-V-54	C2-V-57	C2-V-60	C2-V-63	C2-V-66	C2-V-69	C2-V-72
C3-V-39	C3-V-42	C3-V-45	C3-V-48	C3-V-51	C3-V-54	C3-V-57	C3-V-60	C3-V-63	C3-V-66	C3-V-69	C3-V-72
C1-D-39	C1-D-42	C1-D-45	C1-D-48	C1-D-51	C1-D-54	C1-D-57	C1-D-60	C1-D-63	C1-D-66	C1-D-69	C1-D-72
C2-D-39	C2-D-42	C2-D-45	C2-D-48	C2-D-51	C2-D-54	C2-D-57	C2-D-60	C2-D-63	C2-D-66	C2-D-69	C2-D-72
C3-D-39	C3-D-42	C3-D-45	C3-D-48	C3-D-51	C3-D-54	C3-D-57	C3-D-60	C3-D-63	C3-D-66	C3-D-69	C3-D-72
PBS-PIC	PBS-PIC	PBS-PIC	PBS-PIC	PBS-PIC	PBS-PIC						

Sample abbreviations as in 18.a.

For all rows except the last one, add 85 μL of PBS-PIC and 15 μL of indicated sample.

For the last row, add 100 μL of PBS-PIC.
  e. Using a multi-channel pipette, transfer 35 μL (for KC-lysates) or 15 μL (for C-lysates) from “holding” plate(s) to the pre-filled white 96 well plate(s).
    **CRITICAL:** The volume of the lysates, and the corresponding volumes of PBS-PIC in steps 19.d and 19.e that bring the total volume in each well to 100 μL, need to be optimized for each cell line to achieve fluorescence intensities within the linear range of a plate reader. The fluorescence intensities will depend on the expression levels of Keap1-mCherry or mCherry and may vary between cell lines.
  f. Set up the plate reader. For the Spectramax M2 operated by SoftMax Pro 7.1 (Molecular Devices), select or type in Fluorescence tab within Settings: Excitation 580 nm, Cut off 590 nm, Emission 615 nm (corresponding to the fluorescence spectra of mCherry). In Plate tab, select “96-well standard opaque”.
  g. Place the white plate into the plate drawer, on the top of an adaptor frame for 96-well plates.
  h. Measure the mCherry fluorescence of the entire plate or in selected wells.

#### Quantification and statistical analysis for Keap1-Glow CETSA

**Figure 3:**
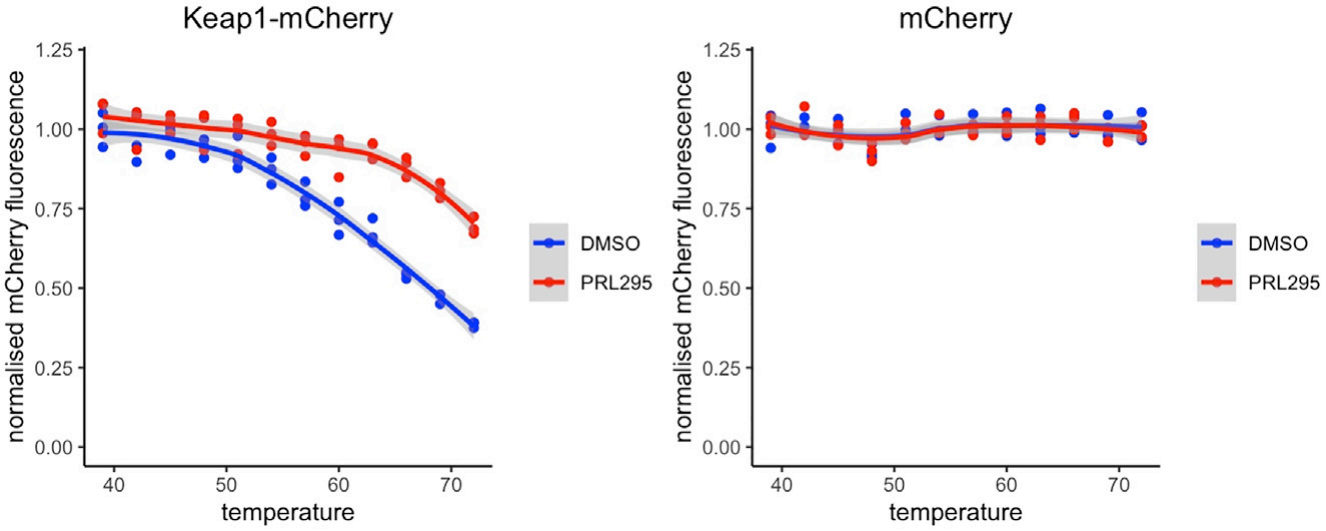
An example of data obtained with Keap1-Glow CETSA (Protocol B) showing that PRL-295 alters the thermostability of Keap1-mCherry, but not free mCherry measured at the same temperature range, in U2OS cell lysates Temperature-dependent aggregation curves in lysates from U2OS cells stably expressing Keap1-mCherry (left) or mCherry (right) pre-treated for 1 h with 15 μM of PRL-295 (red) or the same volume of vehicle (DMSO, blue) are displayed as mCherry fluorescence intensities that remained after removal of precipitated material from lysates heated to the indicated temperatures, normalized to that of vehicle-treated sample heated to 39°C (dots). The smoothing lines that represent local polynomial regressions fitted to the data are added to enhance visualization. Data from Dayalan Naidu et al., 2021.

20. Analyze the data.
  a. Subtract the background calculated as an average fluorescence values of all PBS-PIC samples (“blanks”) in a particular plate, from all the other values in the same plate, to get the background-corrected fluorescence values for the samples.
  b. For each cell type, calculate the average background-corrected fluorescence of vehicle-treated samples that were heated at the lowest temperature (39°C), to get a normalization factor.
  c. Normalize the data by dividing each background-corrected fluorescence value by a normalization factor specific for each cell type. Use the same normalization factor for vehicle and compound-treated cells of the same type.
  d. Plot the normalized data against the heating temperature to visualize the aggregation curve. Optionally, the visualization can be enhanced by fitting the local polynomial regression curve to the data, using R or other suitable software. An example of such plot is shown in Figure 3.

## Expected outcomes

Figure 2 is an example of data obtained with Keap1-CETSA (Protocol A) showing that the Keap1:Nrf2 PPI inhibitor PRL-295 increases the thermostability of Keap1 in intact live HL-60 cells.

Figure 3 is an example of data obtained with Keap1-Glow CETSA (Protocol B) showing that PRL-295 alters the thermostability of Keap1-mCherry, but not free mCherry measured at the same temperature range, in U2OS cell lysates.

Notably, we have observed thermal shifts when cells or cell lysates are incubated with Keap1:Nrf2 PPI inhibitors, but not electrophiles that bind covalently, but reversibly to Keap1. These observations suggest that the assay has the potential to distinguish between the modes of action of Keap1 inhibitors. Further work is required to firmly establish this possibility.

## Limitations

The advantages and limitations of the CETSA methodology in general have been recently reviewed (Seashore-Ludlow et al., 2020). One limitation of the western blot–based CETSA is that its laborious, time-consuming, and semi-quantitative nature does not permit to test exhaustively multiple experimental conditions. This also means that western blot–based Keap1-CETSA cannot be used to screen a large number of compounds. Whereas this limitation is largely overcome by the Keap1-Glow CETSA, one of its limitations is that the levels of expression of the Keap1-mCherry fusion protein are higher than the levels of expression of endogenous Keap1. Furthermore, the intrinsic thermo-stabilizing effect of the mCherry fusion on a target protein in Keap1-Glow CETSA precludes absolute quantifications of Keap1 thermostability and limits its use to applications that assess relative changes in thermostability upon compound binding. Another limitation of the CETSA methodology in general is that it is not possible to distinguish whether a change in the thermal stability of the protein of interest upon compound entry into the cell is a consequence of direct binding to the protein of interest, or a partner of this protein within a protein complex, or even the occurrence of post-translational modification(s). Thus, we recommend the use of this protocol to demonstrate engagement of the Keap1 protein target, in conjunction with other assays, such as assessment of the levels of Nrf2 protein and the expression of its target genes. Finally, this protocol has been established for protein-protein interaction inhibitors of the Keap1:Nrf2 protein complex, and may not be suitable for other Keap1 inhibitors, such as electrophiles that reversibly bind to cysteine sensors in Keap1.

## Troubleshooting

### Problem 1

Uneven heating of lysates or cells during steps 6.c and 6.f of the Keap1-CETSA protocol (Protocol A) and 18.b and 18.d of the Keap1-Glow CETSA (Protocol B).

### Potential solution

Allow the VeriFlex blocks to reach the required temperatures and press pause on the PCR machine to allow time to place the tubes into the appropriate VeriFlex blocks. Alternatively, if there are many samples, the PCR program could be set to 3 min and 15 s at the heat stage to allow for time to place the PCR tubes into the blocks.

### Problem 2

Presence of insoluble aggregates in supernatant.

### Potential solution

After centrifugation (steps 7.e and 7.f of the Keap1-CETSA protocol, Protocol A), ensure to place the microcentrifuge tubes on loosely packed ice or compatible cold blocks to prevent the pellet containing the insoluble aggregates from being dislodged. When transferring the supernatant/soluble fraction, great care must be taken to ensure that the pipette tip does not touch the sides of the microcentrifuge tubes or the pellet.

### Problem 3

**Figure 4:**
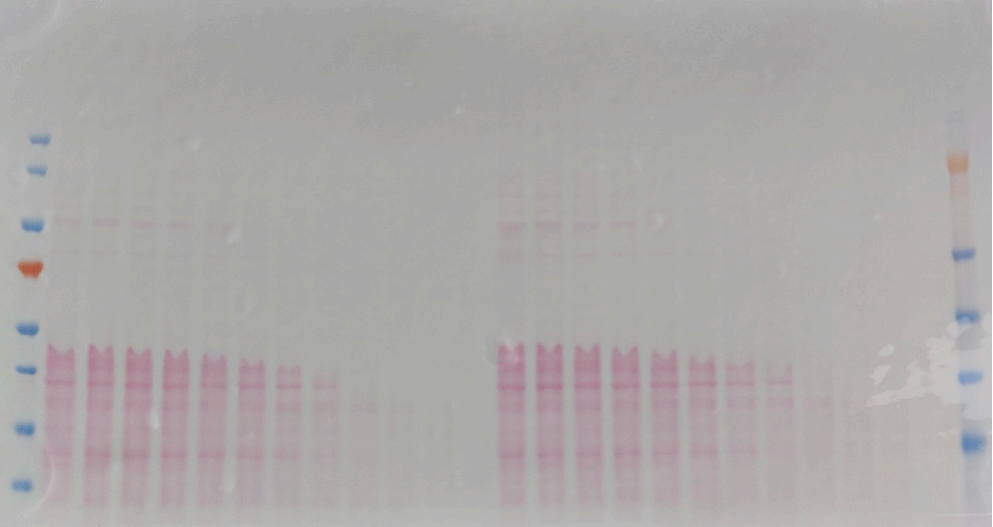
An example of smeared band appearance using the Ponceau S stain following electrophoretic protein separation and membrane transfer

Smeared band appearance (Figure 4).

There have been instances when the proteins migrate aberrantly during electrophoresis, which results in the appearance of smeared bands that migrate below 50 kDa, as shown in this image of a membrane stained with Ponceau S after transfer (step 9.f in the Keap1-CETSA protocol, Protocol A). This effect appears to be cell line specific.

### Potential solution

This problem can be overcome by doubling the concentration of detergent present in the sample loading buffer.

### Problem 4

mCherry fluorescence in one or both cell lines for Keap1-Glow CETSA is too high.

### Potential solution

Prior to the experiment, titrate the volume of the lysate that has high level of fluorescent protein, while keeping the total volume constant by adding PBS or PBS-PIC up to total volume of 100 μL, and establish the volume of the lysate within the linear range of fluorescence measurements. Use the optimized volume of lysate and PBS-PIC in steps 19.d and 19.e of the Keap1-Glow CETSA protocol (Protocol B).

### Problem 5

mCherry fluorescence in one or both cell lines for Keap1-Glow CETSA is too low.

### Potential solution

Increase the volume of the lysate in step 19.e of the Keap1-Glow CETSA protocol (Protocol B) and decrease the volume of PBS-PIC in step 19.d. If the expression levels of fluorescent protein decrease with passaging of the cells, use early passages of the KC-U2OS and/or C-U2OS cells. Increasing the concentration of doxycycline to 1 μg/mL during induction of expression can also increase the expression levels of the fluorescent proteins.

## Resource availability

### Lead contact

Further information and reasonable requests for resources and reagents should be directed to Albena T. Dinkova-Kostova.

### Materials availability

Please contact the lead authors for more information on the reagents.

## Data Availability

The data presented in this article will be shared upon request.
